# Changing Trend in the Antibiotic Resistance Pattern of Klebsiella Pneumonia Isolated From Endotracheal Aspirate Samples of ICU Patients of a Tertiary Care Hospital in North India

**DOI:** 10.7759/cureus.36317

**Published:** 2023-03-17

**Authors:** Abhishek Sharma, Abhishake Thakur, Niketa Thakur, Vineet Kumar, Ankit Chauhan, Neha Bhardwaj

**Affiliations:** 1 Department of Anaesthesia and Critical Care, Shri Guru Ram Das Institute of Medical Sciences and Research, Amritsar, IND; 2 Department of Anaesthesia and Critical Care, Shri Balaji Hospital, Kangra, IND; 3 Department of Radiation Oncology, Shri Guru Ram Das Institute of Medical Sciences and Research, Amritsar, IND; 4 Department of Community Medicine, Indira Gandhi Medical College, Shimla, IND; 5 Department of Cardiac Anaesthesia, UN Mehta Institute of Cardiology and Research Centre, Ahmedabad, IND; 6 Department of Anaesthesia and Critical Care, Dr Rajendra Prasad Government Medical College, Tanda, IND

**Keywords:** multidrug resistant (mdr), endotracheal aspirate, intensive-care unit, antibiotic resistance, multi-drug resistant, klebsiella pneumoniae

## Abstract

Introduction

Klebsiella pneumonia is one of the most prevalent bacteria that cause nosocomial infections, particularly in critically ill patients in the intensive care unit (ICU). Multi-drug-resistant Klebsiella pneumoniae (MDRKP) has become an urgent risk to public health as its prevalence has sharply surged around the globe in recent decades. Therefore, this research was conducted to evaluate shifts over a four-year period in drug susceptibility patterns among Klebsiella pneumoniae isolates from mechanically ventilated intensive care unit patients.

Materials and methods

This is a retrospective observational study conducted in a tertiary care multi-specialty hospital and teaching institute in North India and was approved by the institutional ethics committee. The research comprised Klebsiella pneumoniae isolates from endotracheal aspirates (ETA) of patients on mechanical ventilation admitted to the general intensive care unit (ICU) of our tertiary care facility. The data from January to June 2018 and January to June 2022 were collected. According to the antimicrobial resistance profile of the strains, they were categorized as susceptible, resistant to one or two antimicrobial categories, multidrug-resistant (MDR), extensively drug-resistant (XDR), or pan-drug-resistant (PDR). The criteria for MDR, XDR, and PDR were proposed by the European Centre for Disease Prevention and Control (ECDC). IBM Statistical Package for the Social Sciences (SPSS) for Windows, Version 24.0, Armonk, NY, IBM Corp., was used for data input and analysis.

Results

A total of 82 cases of Klebsiella pneumonia were included in the study. Of these 82 isolates, 40 were isolated over a period of six months from January to June 2018, and the remaining 42 were isolated from January to June 2022. Among the 2018 group, five strains (12.5%) were classified as susceptible, three (7.5%) as resistant, seven (17.5%) as MDR, and 25 (62.5%) as XDR. The highest percentages of antimicrobial resistance in the 2018 group were observed with amoxicillin/clavulanic acid (90%), ciprofloxacin (100%), piperacillin/tazobactam (92.5%), and cefoperazone/sulbactam (95%). In comparison, the 2022 group showed no strain as susceptible; nine strains (21.4%) were classified as resistant; three strains (7%) as MDR; and 30 strains (93%) were classified as XDR.

There was a significant increase in resistance to amoxicillin, from 10% in 2018 to nil in 2022. Overall, the rate of resistant Klebsiella pneumonia (K. pneumonia) increased from 7.5% (3/40) in 2018 to 21.4% (9/42) in 2022, while XDR Klebsiella pneumonia among the mechanically ventilated ICU patients significantly increased from 62.5% (25/40) in 2018 to 71% (30/42) in 2022.

Conclusion

K. pneumoniae antibiotic resistance is a real threat in Asia and requires close monitoring to be controlled. More careful attempts should be made to create a new generation of antimicrobials since the prevalence of resistance to existing medications is rising. Antibiotic resistance should be monitored and reported by healthcare institutions regularly.

## Introduction

Carl Friedlander initially identified Klebsiella pneumoniae in 1882 as a gram-negative, immotile, encapsulated bacterium that was present in the environment. Friedlander's bacillus was the original name before it was changed to Klebsiella in 1886 [1]. It often colonizes the gastrointestinal system and oropharynx of humans (GIT) [1]. Klebsiella pneumonia is implicated in serious healthcare-associated infections, such as pneumonia, bloodstream infections, wound or surgical site infections, and meningitis [2]. Klebsiella pneumonia is one of the most prevalent bacteria that causes nosocomial infections, particularly in critically ill patients in the intensive care unit (ICU) [3]. Multidrug-resistant gram-negative bacterial infections are most often caused by K. pneumoniae strains, according to the literature [4]. Its pathogenicity is caused by the lipopolysaccharide (LPS) layer of the cell envelope and cell wall protein receptors [5]. Multidrug-resistant Klebsiella pneumoniae (MDRKP) has become an urgent risk to public health as its prevalence has sharply surged around the globe in recent decades [6-9]. The term "superbug" has been in use for some time to describe bacterial strains, especially multidrug-resistant and extensively drug-resistant bugs that are immune to most of the available antibiotics [10]. One of the most well-known superbugs to emerge in the last 20 years is Klebsiella pneumonia, which has become multidrug-resistant (MDR) and extensively drug-resistant (XDR) and which may express extended-spectrum beta-lactamases (ESBL), various carbapenemases, and the colistin resistance gene mcr-1 [10]. Ventilator-associated pneumonia (VAP), a hospital-acquired infection, continues to have an impact on the health of 8%-28% of patients receiving mechanical ventilation (MV). MDR and XDR K. pneumoniae-associated VAP infections are linked with increased morbidity, mortality, and prolonged hospital admissions; this has turned into a major issue with a heavy socioeconomic cost [11]. Therefore, to understand the antibiotic sensitivity trends of K. pneumoniae in North India and nosocomial infection control measures and to provide a basis for the selection of appropriate antibiotics, this retrospective study investigated and analyzed the resistance trends of K. pneumoniae over a four-year period in drug susceptibility patterns among isolates from mechanically ventilated intensive care unit patients.

## Materials and methods

This study was conducted at the Shri Guru Ram Das Institute of Medical Sciences and Research, an 800-bed tertiary care multi-specialty hospital and teaching institute in Amritsar, North India. The ethics committee of the institution gave its approval to conduct this study. In this retrospective observational analysis, data from January to June 2018 and January to June 2022 were collected. The research comprised Klebsiella pneumoniae isolates from endotracheal aspirates (ETA) of patients on mechanical ventilation admitted to the general ICU of our tertiary care facility. Only the first isolates were considered, while the same patients' repeated isolates were disregarded. Based on common bacteriological techniques, the isolates were recognized as Klebsiella pneumonia. According to the antimicrobial resistance profile of the strains, they were categorized as susceptible, resistant to one or two antimicrobial categories, multidrug-resistant (MDR), extensively drug-resistant (XDR), or pan-drug-resistant (PDR). The criteria for MDR, XDR, and PDR were proposed by the European Centre for Disease Prevention and Control (ECDC). An isolate is classified as PDR if it is non-susceptible to all specified antimicrobial agents, XDR if it is non-susceptible to at least one agent in all but two or fewer antimicrobial categories, and MDR if it is non-susceptible to at least one agent in at least three antimicrobial categories [12]. IBM Statistical Package for the Social Sciences (SPSS) for Windows, Version 24.0, Armonk, New York, IBM Corp., was used for data input and analysis. In the case of categorical variables, percentages were determined. Two groups were compared using Fisher's exact test or the chi-square test, and statistics were considered significant for all p-values below 0.05.

## Results

A total of 82 cases of Klebsiella pneumonia were included in the study. Of these 82 isolates, 40 were isolated over a period of six months from January to June 2018, and the remaining 42 were isolated from January to June 2022. The age and sex distribution of the study patients have been shown in Table 1. The majority of isolates were in the intermediate age range (31-60 years), where males made up the majority of both demographic groups.

**Table 1:** Gender and age distribution of the study group

	Year 2018 n(%)	Year 2022 n(%)	p-value
Gender			
Male	27 (68%)	26 (61%)	0.765
Female	13 (32%)	16 (39%)	
Age (in years)			
Below or < 50	17 (42.5%)	19 (45%)	0.978
Above or >50	23 (57.5%)	23 (54.7%)	
Total	40	42	

The comparison of the antibiotic sensitivity of Klebsiella pneumonia isolated from ICU patients in 2018 and 2022 is shown in Table 2.

**Table 2:** Antibiotic sensitivity pattern in 2018 and 2022 TMP-SMX: trimethoprim-sulfamethoxazole; Pt: piperacillin-tazobactam; Cfs: cefoperazone-sulbactam

Antibiotic	Year 2018 n(%)	Year 2022 n(%)	p-value
Tigecycline	40 (100%)	42 (100%)	1
Colistin	37 (92%)	12 (28%)	<0.0001
TMP-SMX	15 (38%)	50%	0.277
Minocycline	26.8 (67%)	42 (100%)	0.001
Amoxicillin	4 (10%)	0	0.037
Ceftriaxone	4 (7.5%)	0	0.037
PTZ	3 (7.50%)	1 (3%)	0.361
Cfs	2 (5%)	2 (5%)	1
Cefuroxime	0	2 (5%)	0.154
Imipenem	6 (15%)	3 (7.50%)	0.283
Meropenam	6 (15%)	1 (2.50%)	0.045
Amikacin	7 (17.50%)	5 (12.50%)	0.528
Gentamicin	5 (12.50%)	2 (5%)	0.23
Nalidixic acid	3 (7.50%)	2 (5%)	0.641
Ciprofloxacin	3 (7.50%)	0	0.072

**Figure 1:**
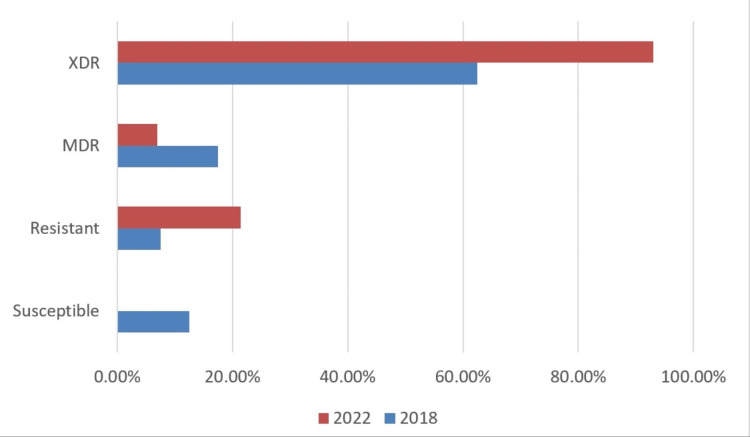
Comparison of resistance profile of klebsiella species between 2018 & 2022 XDR: extensively drug-resistant; MDR: multidrug-resistant The image has been created by the authors.

Among the 2018 group, five strains (12.5%) were classified as susceptible, three strains (7.5%) as resistant, seven strains (17.5%) as MDR, and 25 strains (62.5%) as XDR (Table 3 and Figure 1). The highest percentages of antimicrobial resistance in the 2018 group were observed with amoxicillin/clavulanic acid (90%), ciprofloxacin (100%), piperacillin/tazobactam (92.5%), and cefoperazone sulbactam (95%).

**Table 3:** Antimicrobial resistance classification for Klebsiella species MDR: multidrug-resistant; XDR: extensively drug-resistant, PDR: pan drug-resistant

	Susceptible	Resistant	MDR	XDR	PDR	Total
2018 group	5 (12.5%)	3 (7.5%)	7 (17.5%)	25 (62.5%)	0	40
2022 group	0	9 (21.4%)	3 (7%)	30 (93%)	0	42
p-value	0.048	0.141	0.273	0.532	-	

There was a significant increase in resistance to amoxicillin, from 10% in 2018 to nil in 2022, while Klebsiella remained 100% resistant to ampicillin in both study periods. There was an insignificant decrease in the sensitivity of piperacillin tazobactum in 2022 compared to 2018. The sensitivity of cephalosporins like cefuroxime and cefoperazone sulbactam was found to be less than 5% and statistically insignificant. There was a significant decrease in the sensitivity of the isolates to meropenem in 2022 (2.5%) compared to 2018 (15%). Similarly, there was a highly significant drop in sensitivity to colistin, from 92% in 2018 to 28% in 2022. Sensitivity to trimethoprim-sulfamethoxazole increased from 38% in 2018 to 50% in 2022. Sensitivity to minocycline also increased from 67% in 2018 to 100% in 2022, while Klebsiella remained 100% sensitive to tigecycline in all the tested samples in 2018 and 2022. Overall, the rate of resistant Klebsiella pneumonia increased from 7.5% (3/40) in 2018 to 21.4% (9/42) in 2022, while the rate of XDR-Klebsiella pneumonia increased from 62.5% (25/40) in 2018 to 71% (30/42) in 2022. Although the difference was not significant, a rising trend of resistance was seen. However, the MDR group showed a non-significant decrease in resistance from seven (17.5%) in 2018 to three (7%) in 2022 (p-value = 0.273).

## Discussion

The development of antimicrobial resistance (AMR) is on the rise, particularly among gram-negative microbes, Klebsiella spp. being no exception. Various studies have shown an increased incidence of resistance among Klebsiella species to higher antibiotics. It produces a variety of acute infections, making it problematic, particularly in an intensive care setting. This widespread resistance may be accounted for by the non-judicious use of higher antibiotics without proper sensitivity guidance. Also, India's higher population density may have contributed to the isolation of more multidrug-resistant K pneumoniae species [13]. Klebsiella pneumonia has gained attention in recent years as a "superbug" due to its ability to exhibit resistance to cephalosporins, carbapenems, and colistins. Many different antimicrobial resistance (AMR) plasmids are known to be compatible with Klebsiella, facilitating the bacteria's development of drug resistance. Public health is severely impacted by the worldwide appearance and dissemination of antibiotic-resistant genes in K. pneumoniae isolates, such as extended-spectrum beta-lactamase (ESBL) and carbapenemase genes. This is because carbapenems have long been perceived as the last therapeutic option to treat diseases and infections caused by multidrug-resistant Gram-negative bacteria, which led to the excessive use and misuse of this class of drugs. The speedy worldwide development of K. pneumoniae strains that are resistant to almost all beta-lactam antibiotics, including carbapenems, as shown in this research, demonstrates the organism's quick response to changes in specific environmental stresses. Antimicrobial drug resistance in K. pneumoniae is conferred by genes like plasmid-mediated carbapenemases or enzymes that hydrolyze all beta-lactams [14]. The existence of these carbapenemase-resistant-mediated genes may contribute to the high incidence of carbapenem resistance (imipenem and meropenem) seen in this investigation. The blaKPC genes in K. pneumoniae are mostly carried on plasmids, which provide decreased sensitivity or resistance to almost all beta-lactam antibiotics. The global management of bacterial diseases has a significant problem because of the spread of these resistance genes [15]. As in the study published by Wang et al. [16], K. pneumoniae in our investigation exhibited significant antibiotic resistance. Carbapenems are the first-line treatment for many infections caused by Gram-positive and Gram-negative bacteria since traditionally they have been considered the most effective antibiotics [17]. However, in our study, we discovered that resistance to these drugs was very high and increased steadily over time, from 85% in 2018 to 95% in 2022. Similar to earlier research, K. pneumoniae isolates were found to be totally resistant to ampicillin and amoxicillin [16,18]. Cephalosporins, especially those of the second and third generation, have also been used to treat Klebsiella pneumoniae infection [19]. Compared to 76% of previous studies, we reported almost 100% resistance to second- and third-generation cephalosporins in our investigation [20]. Aminoglycosides have traditionally shown strong effectiveness against Gram-negative bacteria that are clinically significant [21]. In contrast to prior research, where resistance to amikacin and gentamicin was 39.10% and 16.70%, respectively, in our investigation, K pneumoniae isolates were 90% resistant to both drugs [22]. Klebsiella pneumoniae resistance to aminoglycoside antibiotics (amikacin and gentamicin) may be caused by changes in cellular permeability brought on by changes to the AcrAB-TolC and KpnEF efflux pump systems, as well as the disappearance of the putative porin KpnO [23]. We also discovered an alarming spike in colistin resistance, with 72% in the 2022 group against 8% in the 2018 group. This observation may be due to the reintroduction of colistin into clinical practice as a desperate effort to treat multidrug-resistant Gram-negative infections and the indiscriminate usage that's been witnessed in the last two decades. The high prevalence of XDR Klebsiella pneumonia is attributed to the reasons mentioned in Table 4. Remedies to address this issue have been mentioned in Table 5.

**Table 4:** Reasons for the high prevalence of XDR-Klebsiella pneumonia

Possible reasons for the high prevalence of XDR -Klebsiella pneumonia in this study	
1	Most admissions to our ICU are referrals from other healthcare facilities, therefore, prior to exposure to higher antimicrobials
2	Indiscriminate use of antibiotics without culture and sensitivity testing
3	Prolonged use of invasive devices and a long ICU stay
4	Poor infection control strategies and the horizontal spread of resistant strains
5	Prolonged use of broad-spectrum antibiotics

**Table 5:** Remedies to combat antibiotic resistance AMR: antimicrobial resistance, OTC: over the counter

AMR defence measures	
1.	Infection control and prevention
2.	Antimicrobial surveillance
3.	Ban on OTC antibiotics
4.	Hand hygiene
5.	Standard treatment recommendations
6.	Local and national policy
7.	Programmes for education and awareness
8.	New drug development and research

Limitations

The study has certain limitations. This was mainly a retrospective observational study; data were solely taken from microbiology record books, therefore the clinical profile and relevant history of the patient were not taken into consideration. Further, we were unable to identify the origins of these isolates, and the classification of the nature of the infections as community- or hospital-acquired could not be made. Due to a lack of resources, it was not possible to investigate the genomic mechanisms underlying colistin and carbapenem resistance during this study. However, the study adds to the scant literature on the expanding antimicrobial resistance (AMR) issue in this region of the country and demonstrates the difficulties in managing XDR-K. pneumoniae.

## Conclusions

We may draw the conclusion that K. pneumoniae antibiotic resistance is a real threat and requires close monitoring to be controlled. Dealing with "superbugs" like XDR Klebsiella is challenging due to the lack of effective treatment options and the toxicity of last-resort medications. Even though tigecycline and minocycline resistance rates were modest in this research, more careful attempts should be made to create a new generation of antimicrobials since the prevalence of resistance to existing medications is rising. Finally, careful antimicrobial stewardship is essential for preventing the spread of MDR and XDR Klebsiella. Antibiotic resistance should be monitored and reported by healthcare institutions regularly. This will guide practitioners in the prescription of appropriate antimicrobials, the successful treatment of superbugs, and the taking of preventive action against present and future threats.
